# 17β-Estradiol Exacerbated Experimental Occlusal Interference-Induced Chronic Masseter Hyperalgesia by Increasing the Neuronal Excitability and TRPV1 Function of Trigeminal Ganglion in Ovariectomized Rats

**DOI:** 10.3390/ijms22136945

**Published:** 2021-06-28

**Authors:** Yun Liu, Xiao-Xiang Xu, Ye Cao, Si-Yi Mo, Shan-Shan Bai, Ying-Ying Fan, Xiao-Yu Zhang, Qiu-Fei Xie

**Affiliations:** 1 Department of Prosthodontics, Peking University School and Hospital of Stomatology, Beijing 100081, China; liuyun@bjmu.edu.cn (Y.L.); xiaoxiang86@bjmu.edu.cn (X.-X.X.); ye.cao@bjmu.edu.cn (Y.C.); msi715@pku.edu.cn (S.-Y.M.); baisshan@bjmu.edu.cn (S.-S.B.); fan1996@pku.edu.cn (Y.-Y.F.); 2 Center for Oral Functional Diagnosis, Treatment and Research, Peking University School and Hospital of Stomatology, Beijing 100081, China; 3 Central Laboratory, Peking University School and Hospital of Stomatology, Beijing 100081, China

**Keywords:** 17β-estradiol, experimental occlusal interference, hyperalgesia, trigeminal ganglion, neuronal excitability, TRPV1

## Abstract

Pain symptoms in temporomandibular disorders (TMD) predominantly affect reproductive women, suggesting that estrogen regulates pain perception. However, how estrogen contributes to chronic TMD pain remains largely unclear. In the present study, we performed behavioral tests, electrophysiology, Western blot and immunofluorescence to investigate the role and underlying mechanisms of estrogen in dental experimental occlusal interference (EOI)-induced chronic masseter mechanical hyperalgesia in rats. We found that long-term 17β-estradiol (E2) replacement exacerbated EOI-induced masseter hyperalgesia in a dose-dependent manner in ovariectomized (OVX) rats. Whole-cell patch-clamp recordings demonstrated that E2 (100 nM) treatment enhanced the excitability of isolated trigeminal ganglion (TG) neurons in OVX and OVX EOI rats, and EOI increased the functional expression of transient receptor potential vanilloid-1 (TRPV1). In addition, E2 replacement upregulated the protein expression of TRPV1 in EOI-treated OVX rats. Importantly, intraganglionic administration of the TRPV1 antagonist AMG-9810 strongly attenuated the facilitatory effect of E2 on EOI-induced masseter mechanical sensitivity. These results demonstrate that E2 exacerbated EOI-induced chronic masseter mechanical hyperalgesia by increasing TG neuronal excitability and TRPV1 function. Our study helps to elucidate the E2 actions in chronic myogenic TMD pain and may provide new therapeutic targets for relieving estrogen-sensitive pain.

## 1. Introduction

Temporomandibular disorders (TMD) comprise a collection of clinical conditions associated with pain and dysfunction of the masticatory muscles and/or temporomandibular joints (TMJ) [1]. The intensity, incidence and duration of TMD pain are greater in women than men [2]. The prevalence of TMD among women peaks during their reproductive years (aged 20–40 years) and decreases after menopause [3]. Moreover, TMD symptoms and nociceptive thresholds are linked to estrogen levels in women over the menstrual cycle [4,5]. Administration of exogenous estrogens, such as oral contraceptives or hormone replacement therapy (HRT), increases the incidence of TMD pain [6]. Based on these observations, estrogen has been suggested as an important biological factor in the modulation of TMD pain. Nonetheless, the role of estrogen in pain perception is still controversial: some animal studies have indicated its pronociceptive role by increasing responses to mechanical stimuli [7,8,9], while some other results have reported the antinociceptive effect of estrogen [10,11,12]. Therefore, the contribution and the underlying mechanism of estrogen in chronic TMD pain remain to be fully understood. Previously, we established an animal model of dental experimental occlusal interference (EOI) by bonding a crown onto the right maxillary first molar, which induced sustained mechanical hyperalgesia in bilateral masticatory muscles and thus simulating chronic myogenic TMD pain [13]. However, there are no data on whether and how estrogen functions in this model.

Acute and chronic orofacial pain are mediated by primary afferent neurons of the trigeminal ganglion (TG), which may involve modulation of neuronal excitability. Neuron hyperexcitability is characterized by increased numbers of action potentials and decreased thresholds for activation. Evidence has indicated that estrogen receptors (ERs) are present in TG neurons (including small-to medium-diameter nociceptors) [14], and estrogen application in vivo increased the excitability of acutely dissociated primary trigeminal afferents in rats [15], thus making it possible for estrogen to influence nociceptive signaling. It has also been suggested that estrogen might sensitize afferents by affecting the expression and/or function of certain mechanosensitive ion channels [16]. Transient receptor potential vanilloid-1 (TRPV1), which is predominantly expressed in peripheral sensory neurons, can be activated by noxious stimuli, such as heat (>43 °C), acid (pH < 5), some endogenous lipids and pungent chemicals [17], and is involved in pain sensation [18]. Knockout of ERs decreased TRPV1 expression levels in the dorsal root ganglion (DRG) in mice [19], and it has been demonstrated that estrogen facilitates TRPV1 activation in primary sensory neurons [15], suggesting a regulating effect of estrogen in peripheral TRPV1. Derived from the above results, we raised the question of whether estrogen can affect neuronal excitability and TRPV1 function in the TG and thus contribute to pain sensitivity in EOI-induced chronic masseter hyperalgesia.

To address this question, we first determined the role of 17β-estradiol (E2) in EOI-induced chronic masseter hyperalgesia in OVX rats at the behavioral level. Then, we utilized whole-cell patch recordings to investigate the role of E2 in TG neuronal excitability in OVX rats treated with or without EOI. Moreover, we performed molecular biological techniques and intraganglionic injection techniques to explore and further verify TRPV1 function in the TG after E2 application.

## 2. Results

### 2.1. E2 Exacerbated EOI-Induced Masseter Hyperalgesia in a Dose-Dependent Manner in OVX Rats

**Figure 1:**
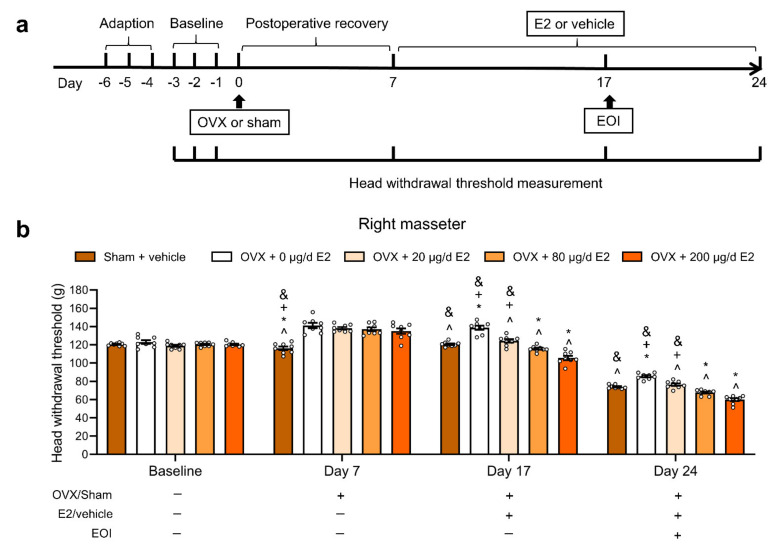
E2 decreased the head withdrawal thresholds in masseter muscles and exacerbated EOI-induced hyperalgesia in OVX rats. (**a**) Schematic diagram of the experimental process and time points of the head withdrawal threshold measurements. (**b**) Head withdrawal thresholds of the right masseter muscle following the procedure shown in Panel A. E2 decreased the head withdrawal thresholds 10 days after consecutive application of E2 and exacerbated EOI-induced decreases in the head withdrawal thresholds of OVX rats in a dose-dependent manner (n = 8 in each group; small circles represent single values). ^ *p* < 0.05 vs. the 0 µg/d E2 group; * *p* < 0.05 vs. the 20 µg/d E2 group; + *p* < 0.05 vs. the 80 µg/d E2 group; and & *p* < 0.05 vs. the 200 µg/d E2 group. Two-way ANOVA followed by Bonferroni’s post hoc tests. OVX, ovariectomized; E2, 17β-estradiol; EOI, experimental occlusal interference.

To evaluate whether E2 exacerbated masseter mechanical hyperalgesia caused by EOI, the head withdrawal thresholds were examined in the sham OVX group and the four OVX groups treated with 0, 20, 80, and 200 μg/d E2, respectively (Figure 1a), and the E2-treated groups have been shown to resemble the physiological E2 levels of rats in menopause, metestrus, proestrus, and pregnancy [9]. As the data of right masseter show in Figure 1, on Day 7 after OVX or sham surgery, there were significant increases in the head withdrawal thresholds of the OVX groups compared with the sham OVX group (*p* < 0.05; Figure 1b), suggesting the physiological E2 level affect mechanical sensitivity. After 10 days of E2 or vehicle replacement (Day 17), the head withdrawal thresholds were correspondingly decreased in the OVX groups treated with increasing doses of E2 (*p* < 0.05; Figure 1b). On Day 7 post EOI (Day 24), E2 dose-dependently exacerbated EOI-induced decreases in the head withdrawal thresholds of OVX rats (*p* < 0.05; Figure 1b). Moreover, the head withdrawal threshold of the sham + vehicle group was lower than that of the OVX + 0 μg/d E2 (vehicle) group and higher than that of the OVX + 200 μg/d E2 group on days 17 and 24 (*p* < 0.05; Figure 1b). The data obtained from left masseter showed the same patterns with right masseter (Supplementary Figure S1a). In addition, the body weights of the OVX groups decreased and the serum E2 levels accumulated with increasing doses of E2 in the OVX groups, confirming the effectiveness of OVX and E2 replacement (*p* < 0.05; Supplementary Figure S1b,c).

### 2.2. E2 Enhanced TG Neuronal Excitability in OVX and OVX EOI Rats

**Figure 2:**
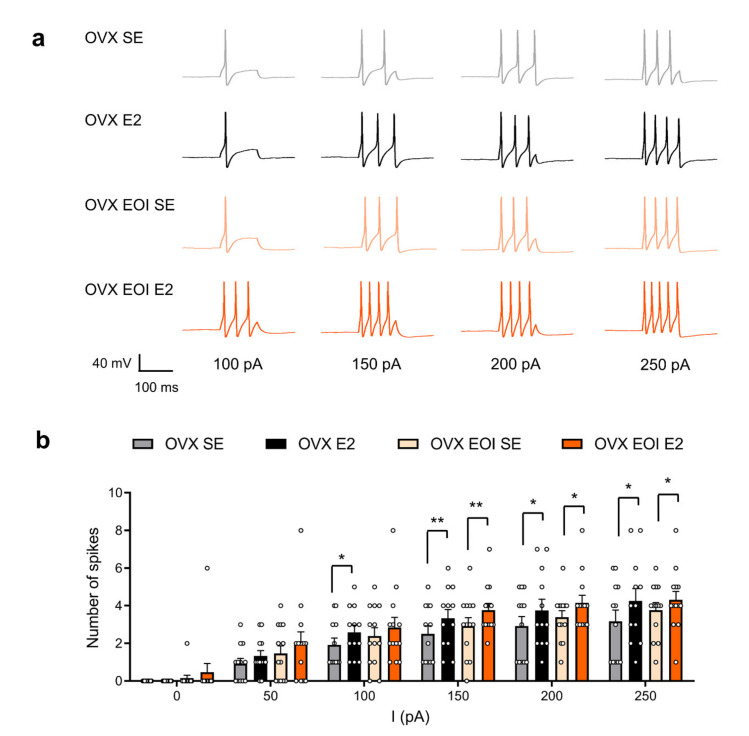
E2 enhanced TG neuronal excitability in OVX and OVX EOI rats. (**a**) Representative action potential traces of TG neurons from OVX and OVX EOI rats induced by different currents (100, 150, 200, and 250 pA) before (SE) and after E2 application (100 nM) for 5 min. (**b**) The number of action potentials in TG neurons (OVX: n = 12 neurons from 4 rats; OVX EOI: n = 13 neurons from 4 rats; small circles represent single values) induced by a series of currents (0, 50, 100, 150, 200, and 250 pA) was increased after E2 application (100 nM). * *p* < 0.05; ** *p* < 0.01; paired *t*-tests. No significant differences were observed between the OVX SE group and OVX EOI SE group or between the OVX E2 group and OVX EOI E2 group; unpaired *t*-tests. OVX, ovariectomized; SE, extracellular solution; E2, 17β-estradiol; EOI, experimental occlusal interference; OVX EOI, OVX rats treated with EOI for 7 days.

To study why masseter mechanical sensitivity was exacerbated by E2 in OVX rats treated with or without EOI, we detected the electrophysiological effect of E2 on TG neuronal excitability in OVX rats and OVX EOI rats (Day 7 post-EOI). TG neurons were injected with a series of 0 to 250 pA currents in 50 pA increments for 100 ms to record action potentials, and the numbers of action potentials before and after extracellular application of E2 (100 nM) for 5 min were compared. Representative tracings of action potentials are shown in Figure 2a. The quantitative results revealed that E2 increased the number of action potentials which were induced by current injection from 100 to 250 pA in TG neurons from OVX rats (150 pA, *p* < 0.01; others, *p* < 0.05; n = 12 neurons from 4 OVX rats). E2 also increased the number of action potentials, especially those in response to injected currents between 150 and 250 pA, in TG neurons from OVX EOI rats (150 pA, *p* < 0.01; others, *p* < 0.05; n = 13 neurons from 4 OVX EOI rats) (Figure 2b). Although the EOI treatment increased the numbers of spontaneous and evoked action potentials slightly, no significant differences were found in the number of action potentials between OVX SE and OVX EOI SE or between OVX E2 and OVX EOI E2 (Figure 2b). The detailed data are shown in Supplementary Table S1. As EOI-induced mechanical hyperalgesia was apparent in OVX rats (Figure 1b,c), suggesting that other mechanisms are involved.

### 2.3. EOI Induced Functional TRPV1 Upregulation in TG Neurons from OVX Rats

**Figure 3:**
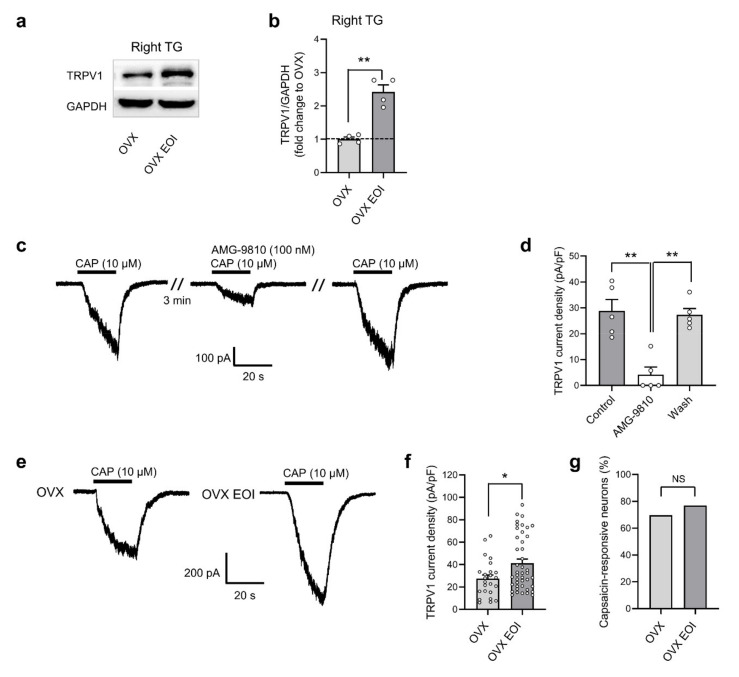
EOI-induced functional TRPV1 upregulation in TG neurons from OVX rats. (**a**) Example Western blots. (**b**) Quantification of the protein level confirmed that EOI increased TRPV1 protein levels in the right TGs. The data were normalized to GAPDH as an internal control, and protein levels were calculated relative to those in OVX rats (n = 4 in each group). ** *p* < 0.01. Unpaired *t*-tests. (**c**) Representative traces of TG neurons in voltage-clamp mode illustrating that the TRPV1 antagonist AMG-9810 almost completely inhibited the capsaicin (10 μM)-induced inward current. (**d**) The current density of TRPV1 in TG neurons was markedly decreased by AMG-9810 (n = 5 neurons; small circles represent single values). ** *p* < 0.01; one-way ANOVA followed by Bonferroni’s post hoc tests. (**e**) Representative traces of TRPV1 currents in TG neurons from OVX rats and OVX EOI rats. (**f**) The TRPV1 current density was higher in neurons from OVX EOI rats (n = 43 neurons from 17 rats; small circles represent single values) than in those from OVX rats (n = 24 neurons from 7 rats; small circles represent single values). * *p* < 0.05; unpaired *t*-tests. (**g**) The percentage of capsaicin-responsive TG neurons in OVX and OVX EOI rats. NS, not significant; unpaired *t*-tests. OVX, ovariectomized; EOI, experimental occlusal interference; CAP, capsaicin; OVX EOI, OVX rats treated with EOI for 7 days.

Because the protein level of TRPV1 was upregulated in TG tissues in male rats after EOI-induced mechanical hyperalgesia [20], we examined whether TRPV1 expression in the TGs of OVX rats was affected by EOI. The commercial TRPV1 antibody (ab203103, Abcam, UK) used in this study was tested for specificity by Western blot and immunofluorescence using TGs of wild-type (WT) and TRPV1 knockout (KO) mice (a generous gift from Dr. Zhuan Zhou from Peking University). Representative protein bands and immunofluorescence staining for TRPV1 were only observed in the WT sample but not in the KO sample, confirming the specificity of the TRPV1 antibody (Supplementary Figure S2). As the protein bands and quantitative results show in Figure 3a,b, compared with those in OVX rats, the protein level of TRPV1 in the right TGs was upregulated in OVX EOI rats (Day 7 post EOI). The data obtained from left TGs showed the same tendency with right TGs (Supplementary Figure S3a). Next, we investigated whether this upregulation was functional in TG neurons. We performed voltage-clamp experiments to record TRPV1 currents activated by the selective TRPV1 agonist capsaicin under a holding potential of −60 mV. Neurons were responsive to capsaicin (10 μM), showing a rapid inward TRPV1 current. The current was almost completely blocked by the TRPV1 antagonist AMG-9810 (100 nM), and it was reversed after washing with extracellular solution (Figure 3c). The current density after AMG-9810 treatment was markedly lower than that under control conditions (*p* < 0.01; Figure 3d), confirming the selectivity of the capsaicin-activated TRPV1 current. Then, we explored whether the TRPV1 current density was increased in TG neurons from OVX rats after EOI. Capsaicin-activated peak currents were recorded in TG neurons isolated from OVX and OVX EOI rats (Figure 3e). Compared with that in the OVX group, the mean current density of TRPV1 was significantly increased in the OVX EOI group (*p* < 0.05; Figure 3f). No difference in the percentage of capsaicin-responsive neurons was found between the OVX and OVX EOI groups (Figure 3g).

### 2.4. E2 Potentiated EOI-Induced TRPV1 Upregulation in TGs

**Figure 4:**
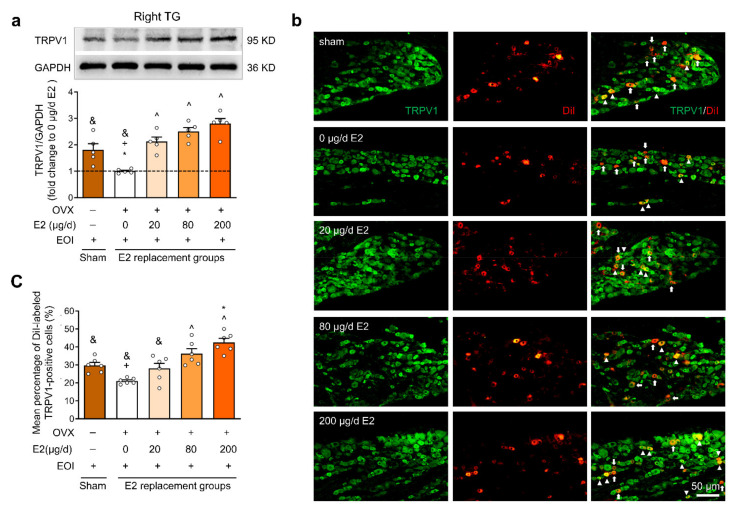
The protein levels of TRPV1 in the TGs were upregulated with increasing doses of E2 in rats that had undergone EOI for 7 days. (**a**) Example Western blots. Quantification of the protein levels confirmed that E2 increased TRPV1 protein levels in the right TGs. The data were normalized to GAPDH as an interval control, and protein levels were calculated relative to those in the 0 µg/d E2 group (n = 5 in each group; small circles represent single values). (**b**) Representative fluorescence photomicrographs of TRPV1 expression (green) in masseter afferent neurons (DiI, red) in the TGs. The long thick arrows indicate DiI-labeled neurons without TRPV1 expression. The triangular arrows indicate double-labeled neurons. (**c**) Quantitative analysis revealed that E2 enhanced the mean percentage of TRPV1-positive neurons in DiI-labeled neurons (n = 6 TGs in each group; small circles represent single values). ^ *p* < 0.05 vs. the 0 µg/d E2 group; * *p* < 0.05 vs. the 20 µg/d E2 group; + *p* < 0.05 vs. the 80 µg/d E2 group; and & *p* < 0.05 vs. the 200 µg/d E2 group. One-way ANOVA followed by Bonferroni’s post hoc tests. OVX, ovariectomized; E2, 17β-estradiol; EOI, experimental occlusal interference.

To investigate whether EOI-induced TRPV1 upregulation was modulated by E2, we performed Western blotting to examine the protein expression of TRPV1 in the bilateral TGs of the sham OVX group and OVX groups treated with various concentrations of E2 (n = 5 for each group) on Day 7 post EOI. The protein expression of TRPV1 in the bilateral TGs was significantly increased in the 80 μg/d and 200 μg/d E2 groups compared with the 0 μg/d E2 group (*p* < 0.05; Figure 4a, Supplementary Figure S3b). To further determine the specific localization of TRPV1 in masseter afferent sensory neurons, DiI retrograde tracing combined with immunofluorescence was used for more detailed observations. Retrograde labeling of masseter afferents was performed in the sham OVX group and OVX groups treated with various doses of E2 (n = 3 for each group) on the day of EOI application. As the protein expression results between right and left TGs are consistent, the data of bilateral TGs were combined statistically analyzed in the immunofluorescence staining experiment (n = 6 TGs per group). TRPV1-positive neurons were found in the V3 and V1/V2 branches of the TGs and predominately expressed in small- to medium-sized cells (Supplementary Figure S4a). DiI-labeled neurons were specifically distributed in the V3 branch of the TGs, and they consisted of small, medium, and large cells (Supplementary Figure S4b). DiI-labeled neurons expressing TRPV1 were identified in all groups (Figure 4b). In the sham OVX group, TRPV1 was expressed in an average of 29.7 ± 2.01% of masseter afferent neurons. E2 replacement increased the percentage of TRPV1-positive masseter afferents in the OVX groups; an increasing trend was observed in the 20 μg/d E2 group (28.1 ± 3.46%), and there were significantly higher percentages in the 80 μg/d (36.3 ± 3.33%) and 200 μg/d (42.8 ± 3.30%) E2 groups than in the 0 μg/d E2 group (21.2 ± 0.76%) (*p* < 0.05; Figure 4c). In brief, these results confirmed that higher E2 levels facilitated TRPV1 expression in TG tissues and masseter afferent neurons of OVX rats treated with EOI. The 80 μg/d E2 dose was used in the subsequent experiment, as it was a lower dose that clearly upregulated TRPV1 expression.

### 2.5. Blocking TRPV1 in TGs Attenuated E2-Mediated Masseter Sensitivity before and after EOI

**Figure 5:**
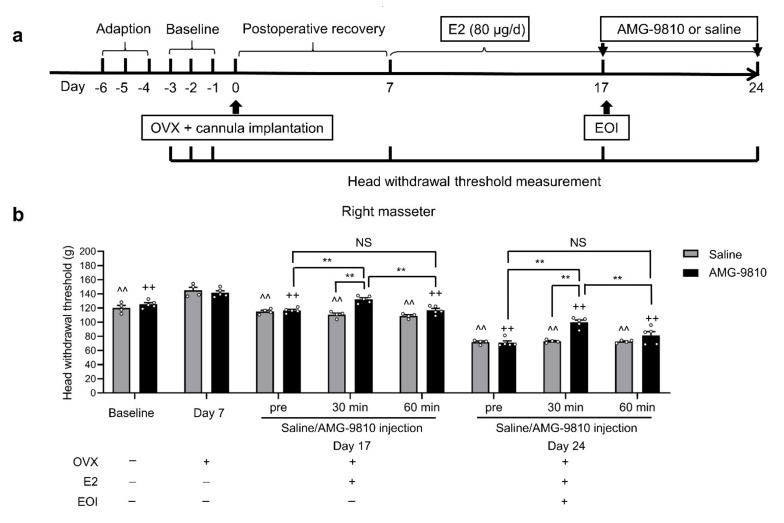
Blocking TRPV1 in TGs attenuated masseter mechanical sensitivity induced by consecutive E2 replacement and EOI in OVX rats. (**a**) Schematic diagram of the experimental process and time points of head withdrawal threshold measurements. Baseline data were obtained by measuring the head withdrawal thresholds of the masseter muscles for three consecutive days before OVX and cannula implantation. The TRPV1 antagonist AMG-9810 or saline was administered to the TGs on Day 10 after E2 replacement (before EOI) and Day 7 post-EOI. Head withdrawal thresholds of the masseter muscles were measured before microinjection as well as 30 and 60 min after intratrigeminal ganglionic microinjection of AMG-9810 (100 nmol in 2 μL, n = 5) or saline (2 μL, n = 4). (**b**) Head withdrawal thresholds of the right masseter muscle were measured following the time course indicated in panel A. Small circles represent single values. ^^ *p* < 0.01, compared with Day 7 in the saline group. ++ *p* < 0.01, compared with Day 7 in the AMG-9810 group. ** *p* < 0.01. Two-way repeated-measures ANOVA followed by Bonferroni’s post hoc tests. OVX, ovariectomized; E2, 17β-estradiol; EOI, experimental occlusal interference.

To further verify whether TRPV1 in the TGs was involved in the facilitatory effect of E2 on EOI-induced masseter hyperalgesia in OVX rats, we microinjected the TRPV1 antagonist AMG-9810 (100 nmol in 2 μL) into the TG to specifically block TRPV1 function in vivo. The placements of the cannulas were confirmed to be within the TG region at the end of the behavioral experiment (Supplementary Figure S5), and the data obtained from rats with injection sites clearly within the TG region were used for the behavioral analysis. As expected, on Day 7 after OVX plus cannula implantation, the head withdrawal thresholds were increased compared with baseline (*p* < 0.05; Figure 5b, Supplementary Figure S6). On Day 10 after E2 replacement (Day 17), the decreases in head withdrawal thresholds were completely reversed 30 min after AMG-9810 microinjection compared with those in the preinjection and saline groups (*p* < 0.05; Figure 5b, Supplementary Figure S6), and no significant effect was observed after 60 min. On Day 7 post-EOI (Day 24), a robust increase in head withdrawal thresholds was observed 30 min after AMG-9810 microinjection compared with those of the preinjection and saline groups (*p* < 0.05; Figure 5b, Supplementary Figure S6), but this increase disappeared after 60 min. There were no significant changes in the head withdrawal thresholds of the masseter muscles 30 or 60 min after saline microinjection compared with those of the preinjection groups (*p* > 0.05; Figure 5b, Supplementary Figure S6). These results demonstrated that TRPV1 in the TGs influenced E2-mediated masseter sensitivity before and after EOI.

## 3. Discussion

Rats used in estrogen research are commonly ovariectomized to ablate the production of cycling hormone, with replacement of E2 exogenously [21]. A series of pain studies have characterized the effects of different modes of E2 administration. Subcutaneous implanted slow-release pellets or silastic capsules containing E2 can be performed more conveniently than daily injections, but there is no direct evidence to support that those methods are more sufficient to match the features of E2 status in vivo. In the present study, we used daily injections of various doses of E2 to produce different levels of circulating E2 in OVX rats. This approach has been widely used because of its stability and reliability. The E2 dosage range used here (0, 20, 80, 200 μg/d) has been shown to resemble the physiological E2 levels of rats in menopause, metestrus, proestrus, and pregnancy [9]. The plasma E2 levels were further determined in our results and were suitable to systematically examine the effect of different E2 levels on EOI-induced chronic masseter mechanical hyperalgesia.

The ability of E2 administration to regulate nociception has been reported in a large number of animal studies but is highly controversial. Some studies have indicated that E2 can alleviate pain [10,11,12], but an increasing number of studies have also suggested that E2 exacerbates pain sensitization through a wide variety of central and peripheral mechanisms [8,9,16,22,23]. In this study, we found that OVX rats showed higher head withdrawal thresholds of bilateral masseter muscles than sham OVX rats, and that regardless of whether EOI was applied, E2 dose-dependently increased masseter mechanical hyperalgesia in OVX rats. These results are in agreement with previous reports showing that mechanical nociception is exacerbated with increasing doses of E2 in rat models of complete Freund’s adjuvant (CFA)-induced TMJ inflammation and glutamate-evoked masseter pain [9,24]. Clinical studies also provided evidence of higher levels of pain in postmenopausal women receiving HRT than in those not receiving HRT [6,25].

The pain information of the orofacial area is transmitted to the central nervous system (CNS) via primary afferent neurons whose cell bodies are located in the TG. Increased neuronal excitability seems to be a key factor for the facilitation of nociception transmission. ERs are expressed in primary afferent neurons, and E2 modulates primary afferent excitability [26,27]. Studies have provided clear evidence that chronic E2 treatment increases the neuronal excitability of rat TMJ afferents and exacerbates the inflammation (CFA)-induced sensitization of these sensory neurons [15] and that acute E2 application at the spinomedullary (V_C_/V_1-2_) region affects the activity of TMJ-responsive neurons [28]. It has also been reported that the estrous cycle modulates the membrane excitability of TG neurons, with a lower action potential (AP) threshold and higher AP height in the proestrus and estrus stages (E2 levels are higher than in the diestrus and metestrus stages) in intact female rats [29]. In the present study, 5 min of E2 (100 nM) treatment increased the number of APs in TG afferents from both OVX rats and OVX EOI rats, indicating that neuronal excitability was rapidly potentiated. Although this application of E2 was not identical to the long-term E2 replacement used in the behavioral experiments, it partially contributed to the increased masseter mechanical sensitivity in both OVX rats and EOI-treated OVX rats following E2 exposure. Several minutes of exposure to 100 nM E2 has also been shown to increase the excitability of hippocampal CA1 pyramidal neurons [30]. As EOI induced significant muscle hyperalgesia but only an increasing tendency (no significant difference) in the excitability of TG neurons, we speculate that neuronal excitability measured in vitro was somewhat different from what happened in vivo, and other mechanisms may contribute to EOI-induced hyperalgesia.

TRPV1 is present in the trigeminal system, including in the peripheral nerve branches, the small to medium sized TG neurons, and the caudal subdivision of the spinal trigeminal nucleus [31,32]. It is well established that TRPV1 is necessary for sensory signal transduction, notably in the sensory detection of noxious mechanical, chemical and thermal stimuli, and plays a critical role in the development of pain hypersensitivity. Studies have shown that TRPV1 is upregulated in the hippocampus as well as synovial tissue in the presence of TMJ inflammation and that TRPV1 antagonists reduce inflammation-associated hyperalgesia [7,33]. Increased protein levels of TRPV1 in the TG were verified in EOI-induced masseter mechanical hyperalgesia in male rats in our previous study [20], and the present study expanded these results in OVX rats. TRPV1 currents could be activated in the presence of the selective agonist capsaicin. In our study, 10 μM capsaicin was used to quantify the fully activated TRPV1 current. This concentration has been used in several studies, and its specificity to the TRPV1 channel has been confirmed in TRPV1-deficient mice [34,35,36]. Although desensitization of the TRPV1 current by topical capsaicin application has been reported in the literature [37,38,39], little or no desensitization of TRPV1 currents with similar capsaicin application has also been reported [40,41,42]. The TRPV1 current did not show much desensitization, which may largely be due to ATP (2 mM) and EGTA (10 mM) in our intracellular solution [42,43]. Our results showed that the current density of TRPV1 in TG neurons was increased in OVX rats after EOI, providing the first electrophysiological evidence for the involvement of peripheral TRPV1 in EOI-induced sensitization. Considering that the activation of TRPV1 can induce an inward current [44] and thus induce neurons to fire action potentials and that E2 can increase the depolarization of TG neuron action potentials, the combined effect may mediate the facilitatory effect of E2 on EOI-induced mechanical sensitivity. While the intrinsic mechanism of pain is very complicated, which may be involved multi-molecular participation, our future work will pay more attention on the function of some other TRP receptors.

E2 binds to ERs and transmits signals by altering the expression of certain proteins in signaling pathways [45,46]. Accumulating evidence has demonstrated that E2 can enhance TRPV1 expression and induce mechanical sensitization [9,33,47,48]. Hippocampal TRPV1 expression and mechanical allodynia were potentiated by E2 following inflammation of the TMJ [9]. Acute and chronic pain coming from the cervix is enhanced by E2 and might be reduced by antagonists of TRPV1 [23]. Some studies have also revealed evidence for E2-induced TRPV1 receptor upregulation and sensitization in a mouse model of neurogenic inflammation and consequent hyperalgesia [47]. Furthermore, ERα and ERβ knockout in mice decreased the expression of TRPV1 in the DRG [19]. Here, we found that higher E2 levels significantly increased the expression of TRPV1 in the TGs and masseter muscle afferents of EOI-treated OVX rats. Although these results did not fully correspond to the dose-dependent effect of E2 on masseter sensitivity, they still suggest that the upregulation of TRPV1 protein expression in TG may participate in the faciliatory effect of E2 on EOI-induced mechanical hyperalgesia. The colocalization of TRPV1 and ERs has been observed in the TG (data not shown), supporting the possibility that E2 directly affects TRPV1 neurons in orofacial pain. E2 signaling in neurons involves genomic signaling pathways related to ERα and/or ERβ and rapid protein kinase signaling by GPR30 in the membrane [46]. However, the subtype of ERs involved in this process needs to be identified in the future.

Numerous studies have demonstrated that TRPV1 antagonists provide substantial relief of hyperalgesia when injected into peripheral terminals, such as masseters [18,20,49], facial skin [50], pulpal tissues [51] and periodontal tissues [52], as well as into the central terminals of nociceptive afferents in the spinal trigeminal nucleus [53,54]. TRPV1 is initially synthesized in the cell bodies of sensory neurons in the TG and then transported to their peripheral and central terminals [55]. There is increasing evidence showing ganglionic mechanisms in pain sensation with TRPV1 upregulation in TG neurons in orofacial neuropathic pain [56] and masseter inflammation [57]. Meanwhile, an injection of the TRPV1 antagonist into the TG is also able to prevent facial hyperalgesia induced by peripheral capsaicin administration [53]. In the present study, we further used AMG-9810, a competitive TRPV1 antagonist with high specificity, to further verify the function of TRPV1 in E2-facilitated mechanical sensitivity with or without EOI. Previous studies have demonstrated that AMG-9810 is effective in alleviating masseter mechanical nociception and hyperalgesia in several models of inflammatory pain [18,49,54]. In our study, pharmacological blockade of TRPV1 was performed via local TG microinjection of AMG-9810. Our results showed that intraganglionic administration of AMG-9810 completely attenuated the facilitatory effect of E2 on nociceptive responses of the masseter muscles of OVX rats before EOI and partially attenuated this effect after EOI. The partial blocking effect of AMG-9810 after EOI may have been due to the involvement of peripheral acid-sensing ion Channel-3 (ASIC3) and ATP-induced purinergic receptors (P2X3 and P2X4) in the development of EOI-induced masseter mechanical hyperalgesia [20,58,59]. Taken together, these results strongly indicate that TRPV1 in TG neurons participates in the facilitatory effect of E2 on masseter muscle mechanical sensitization in the presence or absence of EOI.

## 4. Materials and Methods

### 4.1. Animals

Eighty-nine adult female Sprague–Dawley (SD) rats (initially weighing between 200 and 220 g) were used in this study. Rats were purchased from Vital River Laboratory Animal Technology Co. Ltd. (Beijing, China) and housed in a temperature-controlled room (22 ± 1 °C) under a 12 h light/dark cycle. Food and water were available ad libitum. All experimental procedures were carried out in accordance with the guidelines of the International Association for the Study of Pain (IASP) and were approved by the Institutional Animal Care and Use Committee of Peking University (IACUC; NO. LA2017129, Beijing, China). All efforts were made to reduce the number of animals used and minimize animal suffering.

### 4.2. Ovariectomized (OVX) Surgery

Rats in the OVX group were anesthetized by 1% pentobarbital sodium (50 mg/kg, i.p.), and underwent 2 cm longitudinal dorsal incisions over both flanks under aseptic conditions. The peritoneum was opened through the incision, white adipose tissues were gently grasped until the ovaries and fallopian tubes were exposed, and then the ovaries were removed. The muscle and skin layer were individually sutured. Sham OVX rats underwent similar surgical procedures, but the ovaries were not removed. All rats were administered penicillin to prevent infection and allowed to recover for 7 days. The success of OVX was confirmed by body weight measurements and E2 determination.

### 4.3. Administration of Drugs

The drugs used in this study were 17β-estradiol (E2, E-2758, Sigma, St. Louis, MO, USA), the TRPV1 antagonist AMG-9810 (HY-101736, MedChemExpress, Monmouth Junction, NJ, USA), and the TRPV1 agonist capsaicin (3600376, Sigma, St. Louis, MO, USA). For the behavioral experiments, E2 was dissolved in ethanol and diluted to various concentrations in sterile saline. These drugs were subcutaneously (s.c.) administered daily in the morning in a volume of 200 μL per rat. AMG-9810 was dissolved in dimethylsulfoxide (DMSO) and administered into the TG through a microinjection unit attached to a Hamilton microsyringe. For the patch-clamp experiments, E2, capsaicin, and AMG-9810 were dissolved in DMSO and then diluted with extracellular solution immediately before use. The final concentration of DMSO in the perfusion solution was never greater than 0.1% and thus had no significant effect on the voltage- or current-clamp recordings [60].

### 4.4. Establishment of the EOI-Induced Chronic Masseter Hyperalgesia Model

Detailed procedures for establishing the EOI-induced chronic masseter muscle hyperalgesia model were described previously [13]. In brief, for the EOI group, a metal crown with a thickness of 0.4 mm was cemented onto the upper right first molar of the rat using dental resin bonding material (Panavia F, Kuraray Co., Osaka, Japan). For the control group, the same procedures were performed without the application of EOI.

### 4.5. Retrograde Labeling of Masseter Afferent Neurons

The rats were anesthetized with 1% sodium pentobarbital (50 mg/kg, i.p.) and placed laterally on a heating pad (37 °C) for surgery. The masseter muscle was exposed carefully by incising the superficial skin and separating the fascial tissue. The tracer 1,1′-Dioctadecyl-3,3,3′,3′-tetramethylindocarbo cyanine perchlorate (DiI; Beyotime Biotechnology) was used to identify the primary afferent neurons innervating the masseter muscle in the TG. Ten microliters of DiI (5 mg/mL dissolved in DMSO) was injected slowly using a Hamilton microsyringe (2 μL per site, 5 sites per masseter muscle). To prevent leakage of the retrograde tracer, the injection needle was left in situ for at least 1 min before it was slowly withdrawn. The injection site was covered with petroleum jelly, and the skin layer was sutured carefully. After 7 days, the rats were sacrificed, and the TGs were extracted for immunofluorescence staining. Additionally, the DiI injection sites were confirmed to ensure that the tracer was limited to the masseter muscles.

### 4.6. Guide Cannula Implantation for Intratrigeminal Ganglionic Microinjection

Rats were deeply anesthetized with 1% pentobarbital sodium (50 mg/kg, i.p.) and mounted onto a stereotaxic frame (model 68026, RWD Life Science Company, Shenzhen, China). Two guide cannulas (0.56 mm outer diameter and 0.38 mm inner diameter) were implanted at the following coordinates, as determined by the rat brain atlas: 3.5 mm posterior to the bregma, 3.6 mm lateral to the midline, and 9 mm ventral to the skull surface (Supplementary Figure S5a). The guide cannulas were anchored to the skull using stainless-steel screws and self-curing dental acrylic resin. A stainless-steel stylet (11 mm in length and 0.3 mm in diameter) was inserted into the cannula to reduce the incidence of obstruction and infection. AMG-9810 (100 nmol in 2 μL) or saline was microinjected into the TG using a microinjection unit that extended 2 mm beyond the tip of the guide cannula. The application of AMG-9810 were selected according to the specificity and effectiveness from previous reports [49,61].

The microinjection unit was attached to a Hamilton microsyringe (RWD Life Science Company) via PE-10 polyethylene tubing, and an infusion pump was programmed to deliver a volume of 2 μL to the TG over a period of 1 min. The needle was kept in place for at least 1 min before retraction to allow the reagent to diffuse sufficiently and effectively. The injection sites were inspected by visual examination and direct blue (2 μL) microinjection upon completion of the behavioral experiment (Supplementary Figure S5c). Only data obtained from rats with injection sites clearly within the TG were used for the behavioral analysis.

### 4.7. Evaluation of the Masseter Mechanical Sensitivity

Mechanical sensitivity in the masseter was tested as the head withdrawal threshold using a modified electronic von-Frey aesthesiometer (BIO-EVF3, Bioseb, Vitrolles, France) as previously reported [13]. Briefly, rats were acclimated to the testing environment for 30 min for three consecutive days before baseline evaluation. The rat was free to withdraw its head from the progressively increasing mechanical stimulus over the belly region of the masseter. The applied force in grams was automatically recorded when the head was withdrawn. The head withdrawal thresholds were averaged based on five measurements per masseter. The baseline mechanical threshold was determined over three continuous days. All data reported in the behavioral experiment were evaluated by a trained tester with blind method.

### 4.8. Behavioral Experiments

#### 4.8.1. Effects of E2 on EOI-Induced Masseter Hyperalgesia

This experiment was designed to investigate whether E2 can enhance EOI-induced chronic masseter hyperalgesia in rats. Forty rats were randomly assigned to 5 groups (n = 8 per group): the sham OVX group and 4 OVX groups treated daily with 0, 20, 80 or 200 μg E2. The sham OVX group received daily subcutaneous injections of the same volume of vehicle (10% ethanol in sterile saline). All rats underwent EOI after 10 days of E2 or vehicle application. Head withdrawal thresholds of the bilateral masseter muscles were measured on preapplication Days 1, 2, and 3 (baseline); Day 7 after OVX or sham surgery (before E2 replacement); Day 10 after E2 or vehicle replacement (before EOI application); and Day 7 after EOI application.

#### 4.8.2. Effects of the TRPV1 Antagonist AMG-9810 on E2-Potentiated EOI-Induced Masseter Hyperalgesia

This experiment was designed to assess whether the TRPV1 antagonist AMG-9810 can attenuate EOI-induced masseter muscle hyperalgesia in E2-treated rats. Baseline masseter muscle mechanical sensitivity was evaluated before OVX and cannula implantation. After 7 days of postoperative recovery, 80 μg/d E2 replacement began. Saline or the TRPV1 antagonist AMG-9810 (100 nmol in 2 μL) was administered to the bilateral TGs on Day 10 after E2 replacement (before EOI application) and Day 7 after EOI application. Head withdrawal thresholds of bilateral masseter muscles were measured before microinjection as well as 30 and 60 min after intratrigeminal ganglionic microinjection of saline (n = 4) or AMG-9810 (n = 5).

### 4.9. E2 Determination

The rats were sacrificed with an overdose of pentobarbital sodium (100 mg/kg, i.p.) at the end of the experiment. Blood samples were collected from the abdominal aorta and then centrifuged at 3000 rpm for 20 min to extract the serum. The plasma E2 levels were determined by chemiluminescence using an Access Immunoassay system (UniCel DxI 800).

### 4.10. Western Blot Analysis

After administering pentobarbital sodium (100 mg/kg, i.p.), rats were deeply anesthetized. The bilateral TGs were harvested and homogenized in ice-cold RIPA lysis buffer supplemented with protease inhibitor cocktail (Huaxingbio Science, Beijing, China). The tissue lysates were centrifuged at 4 °C for 20 min at 14,000 *g*. The supernatants containing protein lysates were collected. Protein concentrations were then quantified with a BCA protein assay kit (Beyotime Biotechnology, Shanghai, China). Fifty μg total protein samples were subjected to 8% SDS-PAGE gels, separated by electrophoresis in Tris-glycine buffer at 100 V, and then transferred to polyvinylidine difluoride (PVDF) membranes in transfer buffer. The membranes were blocked in 5% bovine serum albumin (BSA) in TBS-T buffer for 2 h at room temperature and then incubated overnight at 4 °C with a primary antibody against TRPV1 (1:1000 dilution; Abcam, UK). After washing with TBST, the membranes were incubated with a secondary antibody conjugated to horseradish peroxidase (HRP) (1:10,000 dilution; Zhong Shan Golden Bridge, Beijing, China) for 1 h. Peroxidase activity was detected using an ECL chemiluminescent kit (Beyotime Biotechnology), and images were collected with a luminescent image analyzer (Fusion FX, Vilber Lourmat, France). Anti-GAPDH (1:2000 dilution; Zhong Shan Golden Bridge) was used as a protein internal control. The protein expression was quantified with ImageJ 1.38 software (National Institutes of Health, Bethesda, MD, USA) and normalized to GAPDH.

### 4.11. Immunofluorescence Staining

Rats were deeply anesthetized and transcardially perfused with 300 mL body-temperature physiological saline followed by 200 mL 4% ice-cold paraformaldehyde (Sigma-Aldrich) in 0.1 M phosphate buffer saline (PBS, pH 7.4). TGs were isolated immediately and postfixed overnight at 4 °C in 4% paraformaldehyde/0.1 M PBS and then dehydrated in a 30% sucrose solution (in 0.1 M PBS) for several days. The TGs were embedded and sectioned transversely into 10 μm thick sections on a cryostat. After blocking the sections with 10% goat serum and 0.3% Triton-X 100 (Sigma) in PBS for 1 h, sections were incubated with a mouse monoclonal anti-TRPV1 antibody (1:500 dilution; Abcam, Cambridge, UK) overnight at 4 °C, followed by fluorescein isothiocyanate (FITC)-conjugated goat anti-mouse IgG (1:100 dilution; Zhong Shan Golden Bridge, Beijing, China) for 1.5 h at room temperature. Five stained sections of each TG were observed with a fluorescence microscope (BX51; Olympus, Tokyo, Japan). Images were examined and captured using a charge-coupled device camera with two filters for DiI and FITC separately (DP71; Olympus, Tokyo, Japan). The microscope settings remained consistent throughout the image capture process.

Immunopositive neurons were counted with Image-Pro Plus v6.0 software (Media Cybernetics, Rockville, MD, USA). The percentage of TRPV1-positive neurons in masseter afferent neurons was calculated by the following formula: (the number of neurons dually labeled with TRPV1 and DiI)/(the number of neurons labeled with DiI) × 100%. The researcher who performed this analysis was blinded to the grouping.

### 4.12. Isolation of TG Neurons

To investigate the change in TG neuronal excitability after E2 treatment and the role of TRPV1 in EOI-induced masseter hyperalgesia, patch-clamp recordings of neurons from OVX rats (n = 7) and OVX rats treated with EOI for 7 days (OVX EOI; n = 17) were performed. The TGs were dissected and treated with Dulbecco’s modified Eagle’s medium (DMEM) containing 1 mg/mL collagenase and 0.3 mg/mL trypsin for 40 min at 37 °C. After digestion, the samples were collected and centrifuged at 800 rpm for 3 min. The supernatants were discarded, and the pellets were suspended in DMEM containing 10% horse serum. Then, the cells were plated on poly-L-lysine-coated glass coverslips (12 mm in diameter) and incubated in a humidified atmosphere of 5% CO_2_ at 37 °C. TG neurons were used for recordings within 2–6 h after dissociation.

### 4.13. Patch-Clamp Recording

Whole-cell patch-clamp recordings were performed under current or voltage-clamp conditions at room temperature (22–24 °C) using a HEKA EPC10 amplifier (HEKA Electonik). Patch pipettes with a resistance between 2 and 2.5 MΩ were made from hard borosilicate glasses with a two-step vertical puller. Electrophysiological signals were recorded from small- to medium-sized TG neurons with a membrane capacitance between 15–40 pF. The bath (extracellular) solution contained (in mM) 150 NaCl, 5 KCl, 2.5 CaCl_2_, 1 MgCl_2_, 10 HEPES, and 10 glucose, and the pH was adjusted to 7.4 with NaOH. The pipette (intracellular) solution contained (in mM) 100 KCl, 42 KOH, 10 HEPES, 2 CaCl_2_, 2 MgCl_2_, 10 EGTA, 2 Mg-ATP, and 2 Na-GTP, and the pH was adjusted to 7.2 with KOH. The fast electrode capacitance was first compensated before breaking into the cell. After whole-cell configuration was achieved, capacitive transients were cancelled by using an automatic compensation function and monitored periodically. The access (series) and input resistances of all cells were monitored and recorded periodically throughout the experiment. Only cells with access resistances less than 20 MΩ and within a 20% change were included for analysis. Drugs were applied extracellularly using a pressure-driven multichannel system (InBio, Wuhan, China).

For current-clamp mode, the cells were injected with a series of 100 ms currents from 0 to 250 pA in 50 pA increments (step) to record evoked action potentials before and after E2 (100 nM) treatment for 5 min. For voltage-clamp mode, the cells were constantly clamped at a holding potential of −60 mV, and the TRPV1 channel current was induced by capsaicin (10 μM) for 20 s. The TRPV1 antagonist AMG-9810 (100 nM) and agonist capsaicin (10 μM) were coapplied extracellularly to inhibit TRPV1 during the measurement. Capsaicin was applied at intervals of 3–5 min to record TRPV1 currents repeatedly. To study and confirm the blocking effect of AMG-9810 on capsaicin-induced currents, cells without marked desensitization that recovered to its basal level after washing were selected for analysis. Additionally, TG neurons in each dish were treated with capsaicin only once in subsequent experiments to avoid the possible acute desensitization of TRPV1 channels to capsaicin.

### 4.14. Statistical Analysis

SPSS 20.0 (SPSS, Chicago, IL, USA) was used to perform statistical analyses. The results are expressed as the mean ± standard error of the mean (SEM). Differences in data between more than two groups were analyzed by ANOVA followed by Bonferroni’s post hoc tests for multiple comparisons. The effects of E2 and EOI on TG neuronal excitability were assessed using *t*-tests. The number of action potentials in TG neurons from OVX and OVX EOI rats before and after E2 treatment were analyzed using paired *t*-tests. The number of action potentials and TRPV1 current densities in TG neurons and the protein levels of TRPV1 in the bilateral TGs were compared between OVX and OVX EOI rats using unpaired *t*-tests. *p* < 0.05 was considered to indicate a significant difference.

## 5. Conclusions

In summary, our study provides evidence for the exacerbating effect of E2 in EOI-induced chronic masseter hyperalgesia by increasing the neuronal excitability and TRPV1 expression in TG neurons. The upregulation of TRPV1 by E2 in EOI model may explain the greater mechanical sensitivity in reproductive age women in clinical occlusion-related pain, and might offer clues for promising therapeutic targets for E2-sensitive chronic orofacial pain.

## Data Availability

Data is contained within the article or Supplementary Materials.

## Supplementary Materials

Supplementary Materials can be found at https://www.mdpi.com/article/10.3390/ijms22136945/s1. Figure S1: head withdrawal thresholds of the left masseter muscle, weight and plasma E2 changes after treatment with different doses of E2. Figure S2: validation of TRPV1 antibody using receptor-deficient mice. Figure S3: example Western blots and quantitative results in the left TG. Figure S4: immunofluorescence of the expression of TRPV1 in masseter afferent neurons. Figure S5: implantation of the guide cannula for intratrigeminal ganglionic microinjection and anatomical confirmation of the injection site. Figure S6: head withdrawal thresholds of the left masseter muscle were measured following the time course indicated in Panel A in Figure 5a. Table S1. the mean number of spikes in TG neurons from OVX and OVX EOI rats induced by currents before (SE) and after E2 application.

## Abbreviations

OVX	Ovariectomized
E2	17β-estradiol
EOI	Experimental occlusal interference
TRPV1	Transient receptor potential vanilloid-1
WT	Wild-type
KO	Knockout
DMEM	Dulbecco’s modified Eagle’s medium
DMSO	Dimethylsulfoxide
DiI	1,1′-Dioctadecyl-3,3,3′,3′-tetramethylindocarbo cyanine perchlorate
FITC	Fluorescein isothiocyanate
SD	Sprague–Dawley
AP	Action potential
CFA	Complete Freund’s adjuvant
TMD	Temporomandibular disorders
TMJ	Temporomandibular joints
