# Supplementary material for: 17β-Estradiol Exacerbated Experimental Occlusal Interference-Induced Chronic Masseter Hyperalgesia by Increasing the Neuronal Excitability and TRPV1 Function of Trigeminal Ganglion in Ovariectomized Rats
Source: Int J Mol Sci. 2021 Jun 28;22(13):6945. doi: 10.3390/ijms22136945 (PMC8269106; doi:10.3390/ijms22136945)
Supplement: Supplementary file 1 [file ijms-22-06945-s001.zip › ijms-1234683-supplementary.pdf]

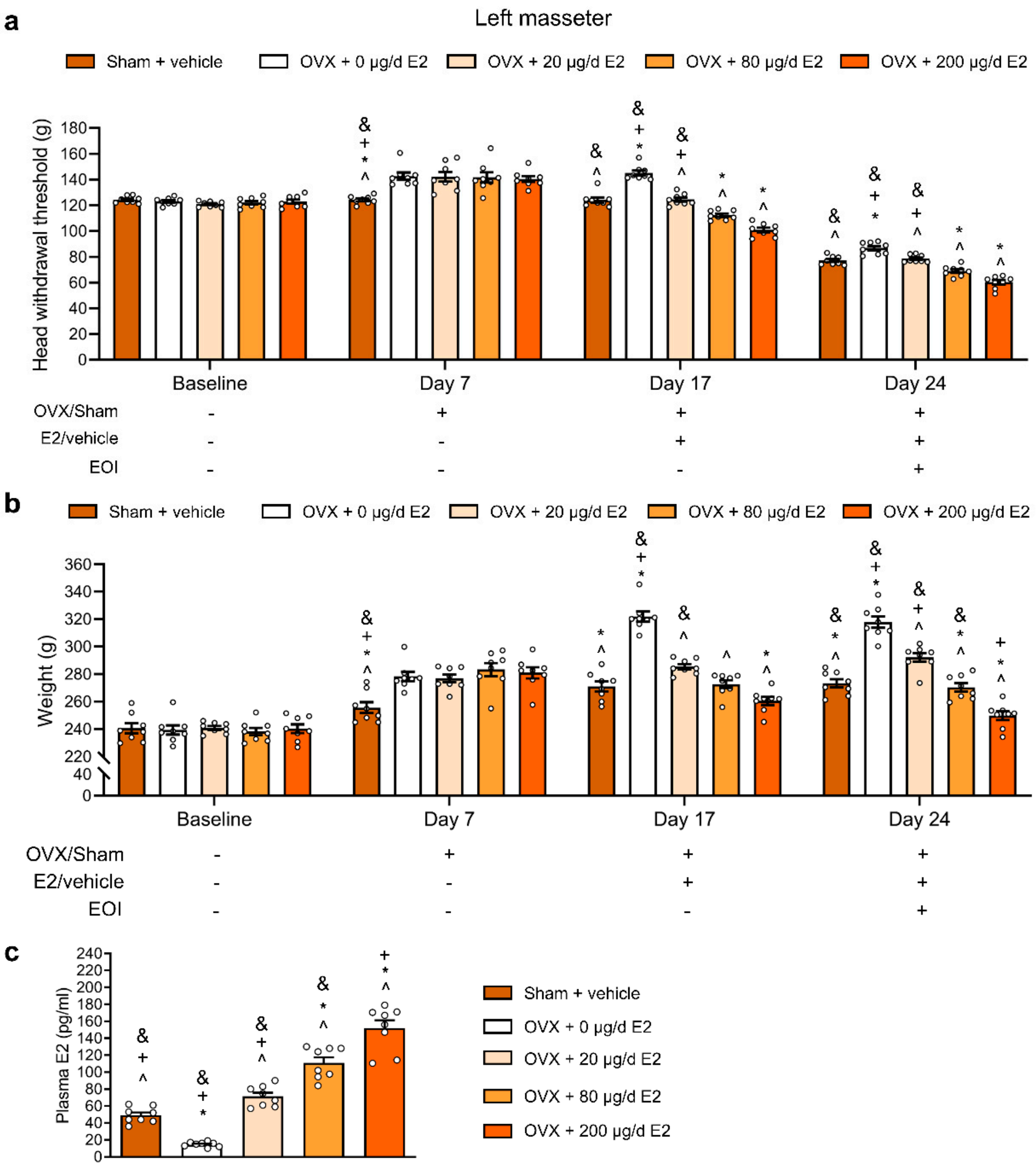

Figure S1. Head withdrawal thresholds of the left masseter muscle, weight and plasma E2 changes after treatment with different doses of E2. (**a**) Head withdrawal thresholds of the left masseter muscle following the procedure shown in panel A in Figure 1. (**b**) The weights of the OVX rats decreased as the dose of E2 increased (n=8 in each group). (**c**) Plasma levels of E2 in OVX rats increased as the dose of E2 increased (n=8 in each group). ^: $p < 0.05$ vs. the 0 μg/d E2 group; *: $p < 0.05$ vs. the 20 μg/d E2 group; +: $p < 0.05$ vs. the 80 μg/d E2 group; &: $p < 0.05$ vs. the 200 μg/d E2 group. Two-way repeated-measures ANOVA or one-way ANOVA, followed by Bonferroni's *post hoc* tests. OVX, ovariectomized; E2, 17β-estradiol; EOI, experimental occlusal interference.

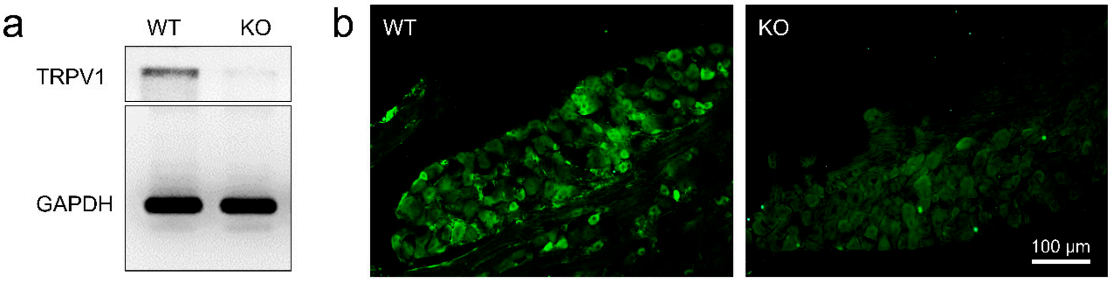

Figure S2. Validation of TRPV1 antibody using receptor-deficient mice. Representative protein band (**a**) and immunofluorescence staining (**b**) for TRPV1 protein expression in the TG from wild-type (WT) mice and TRPV1 knockout (KO) mice. TRPV1 was only observed in the WT sample but not in the KO sample, confirming the specificity of the TRPV1 antibody.

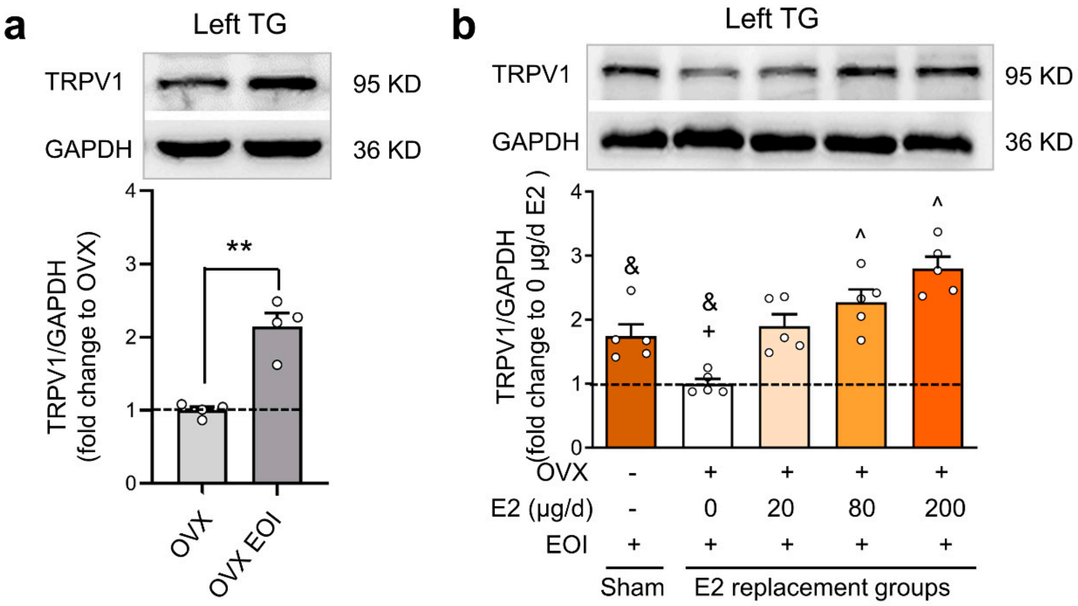

Figure S3. Example western blots and and quantitative results in the left TG. (**a**) Quantification of the protein levels confirmed that EOI increased TRPV1 protein levels in the left TGs. The data were normalized to GAPDH as an internal control, and protein levels were calculated relative to those in OVX rats (n = 4 in each group). **: $p < 0.01$. Unpaired *t*-tests. (**b**) Quantification of the protein levels confirmed that E2 increased TRPV1 protein levels in the right TGs. The data were normalized to GAPDH as an interval control, and protein levels were calculated relative to those in the 0 μg/d E2 group (n = 5 in each group). ^: $p < 0.05$ vs. the 0 μg/d E2 group; *: $p < 0.05$ vs. the 20 μg/d E2 group; +: $p < 0.05$ vs. the 80 μg/d E2 group; &: $p < 0.05$ vs. the 200 μg/d E2 group. One-way ANOVA followed by Bonferroni's *post hoc* tests. OVX, ovariectomized; OVX EOI, OVX rats treated with EOI for 7 days; E2, 17β-estradiol; EOI, experimental occlusal interference.

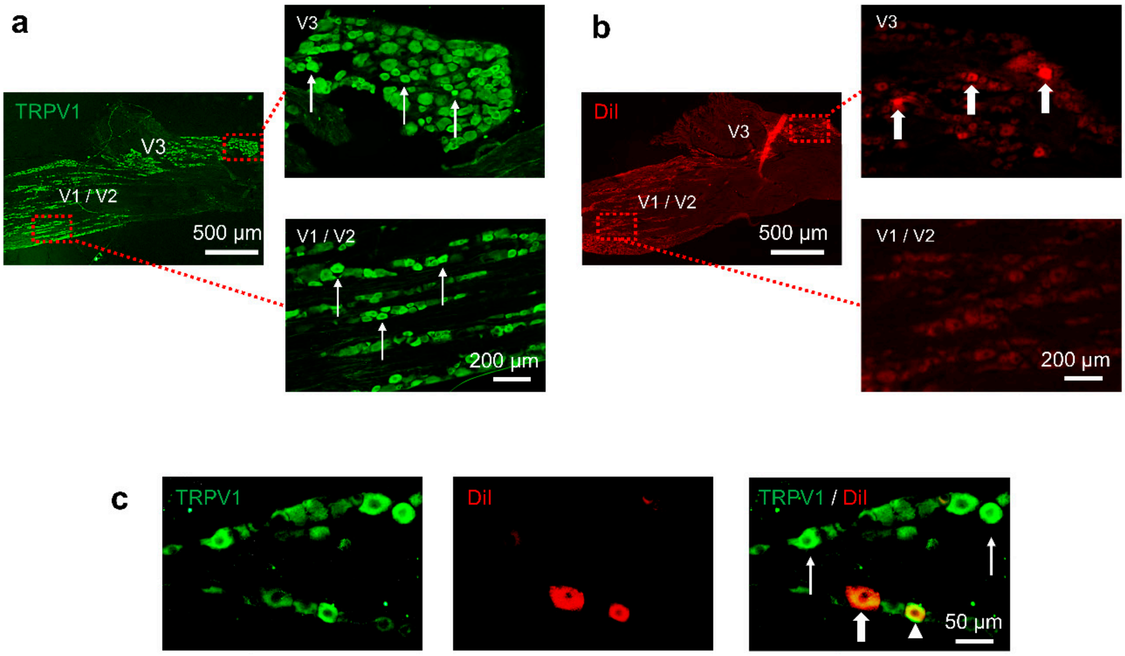

Figure S4. Immunofluorescence of the expression of TRPV1 in masseter afferent neurons. (**a**) Fluorescence photomicrographs of the expression of TRPV1 (green) in the TG. (**b**) Masseter afferent neurons (red) in the TG were labeled by injecting DiI into the masseter muscle. (**c**) Representative fluorescence photomicrographs of the expression of TRPV1 in masseter afferent neurons. The long thin arrows indicate TRPV1-positive neurons, and the long thick arrows indicate DiI-labeled neurons not expressing TRPV1. The triangular arrows indicate double-labeled neurons.

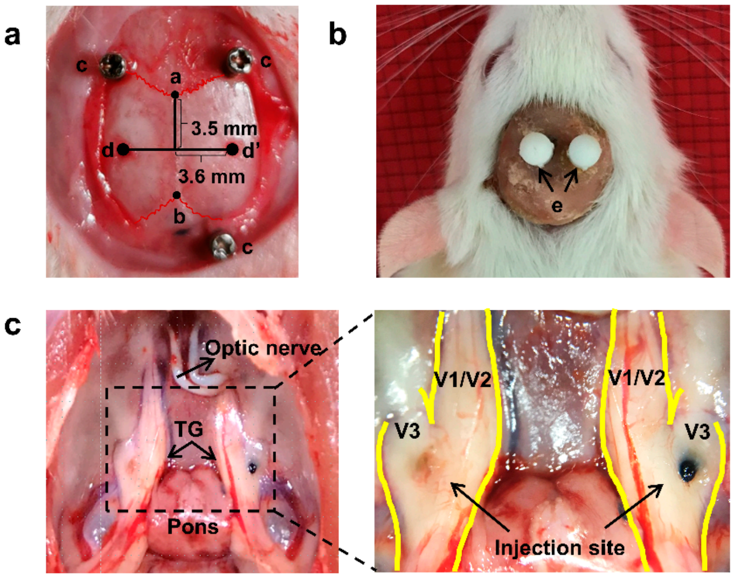

Figure S5. Implantation of the guide cannula for intratrigeminal ganglionic microinjection and anatomical confirmation of the injection site. (**a**) The coordinates used for implantation were determined based on anatomical landmarks on the skull. a: bregma; b: lambda; c: screws; d; d′: entrance of the guide cannula. (**b**) The fixed device used for microinjection remained in good condition until the end of the experiment. e: caps of the guide cannulas. (**c**) The anatomical location of the injection site was confirmed. The drug injection sites were confirmed by visual examination (left TG) and direct blue injection (right TG) upon completion of the experiment.

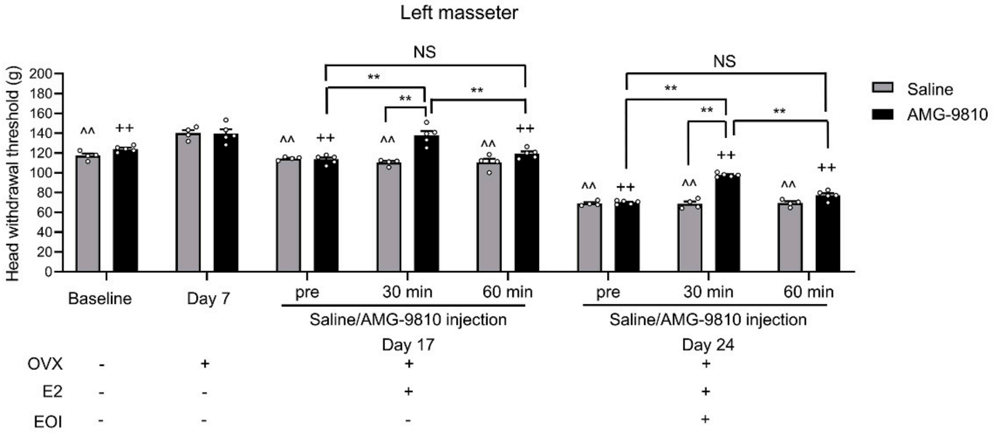

Figure S6. Head withdrawal thresholds of the left masseter muscle were measured following the time course indicated in panel A in Figure 5a. ^^: $p < 0.01$, compared with day 7 in the saline group. ++: $p < 0.01$, compared with day 7 in the AMG-9810 group. **: $p < 0.01$. Two-way repeated-measures ANOVA followed by Bonferroni's *post hoc* tests. OVX, ovariectomized; E2, 17β-estradiol; EOI, experimental occlusal interference.

Table S1. The mean number of spikes in TG neurons from OVX and OVX EOI rats induced by currents before (SE) and after E2 application.

	OVX (n=4 rats)		OVX EOI (n=4 rats)	
**Currents (pA)**	**SE (n=12)**	**E2 (n=12)**	**SE (n=13)**	**E2 (n=13)**
0	0.0 ± 0.0	0.0 ± 0.0	0.2 ± 0.2	0.5 ± 0.5
50	0.9 ± 0.3	1.3 ± 0.3	1.5 ± 0.4	2.0 ± 0.6
100	1.9 ± 0.4	2.6 ± 0.4*	2.4 ± 0.4	2.8 ± 0.5
150	2.5 ± 0.4	3.3 ± 0.5**	2.9 ± 0.4	3.8 ± 0.4^^
200	2.9 ± 0.5	3.8 ± 0.6*	3.4 ± 0.3	4.2 ± 0.4^
250	3.2 ± 0.6	4.3 ± 0.7*	3.8 ± 0.4	4.3 ± 0.5^

Values are presented as means ± SEM. * The OVX E2 group was significantly different from the OVX SE group (*: $p < 0.05$; **: $p < 0.01$; paired *t*-tests). ^ The OVX EOI E2 group was significantly different from the OVX EOI SE group (^: $p < 0.05$; ^^: $p < 0.01$; paired *t*-tests). There were no significant differences between the OVX SE group and the OVX EOI SE group or between the OVX E2 group and the OVX EOI E2 group (paired *t*-tests). OVX, ovariectomized; SE, extracellular solution; E2, 17β-estradiol; EOI, experimental occlusal interference; OVX EOI, OVX rats treated with EOI for 7 days.
